# Persistent double strand break accumulation does not precede cell death in an Olaparib-sensitive BRCA-deficient colorectal cancer cell model

**DOI:** 10.1590/1678-4685-GMB-2019-0070

**Published:** 2019-12-13

**Authors:** Natalia Soledad Paviolo, María Belén de la Vega, María Florencia Pansa, Iris Alejandra García, Nicolás Luis Calzetta, Gastón Soria, Vanesa Gottifredi

**Affiliations:** 1 Fundación Instituto Leloir-Instituto de Investigaciones Bioquímicas de Buenos Aires. Buenos Aires, Argentina; 2 Centro de Investigaciones en Bioquímica Clínica e Inmunología, CIBICI-CONICET. Córdoba, Argentina; 3 Departamento de Bioquímica Clínica. Facultad de Ciencias Químicas, Universidad Nacional de Córdoba. Córdoba, Argentina

**Keywords:** GammaH2AX, alternative end joining, non-homologous end joining, homologous recombination, PARP

## Abstract

The poly (adenosine diphosphate (ADP)-ribosyl) polymerase inhibitors (PARPi) selectively kill cancer cells with BRCA1 or BRCA2 (BRCA)-mutations. It has been proposed that cell death induction after PARPi depends on unrepaired double strand breaks (DSBs) that accumulate due to the homologous recombination deficiency of BRCA-mutated cells. Such accumulation of DSBs is inferred mainly from the high levels of DNA damage markers like phosphorylated histone H2AX. Herein, we developed a model of isogenic cell lines to show that depletion of BRCA causes PARPi-triggered cell death, replication stress (phosphorylated-H2AX and 53BP1 foci), and genomic instability. However, persistent DSBs accumulation was not detected under the same experimental conditions. Hence, at least in this cellular model, the trigger for cell death in PARPi-treated BRCA-depleted samples is not the accumulation of unrepaired DSBs. Instead, cell death better correlates with a rapid and aberrant resolution of DSBs by error-prone pathways that leads to severe chromosomic aberrations. Therefore, our results suggest that in PARPi-treated BRCA-deficient cells, chromosome aberrations may dually trigger both genomic instability and cell death.

## Introduction

Homologous recombination (HR)-deficiency leads to genomic instability due to the shift from “error-free” to “error-prone” DNA repair pathways (Prakash *et al.*, 2015; Talens *et al.*, 2017). HR-deficiency is, therefore, a driver of tumorigenesis as demonstrated in cancer cells deficient in BRCA1- and BRCA2 (BRCA) expression or functions (Fackenthal and Olopade, 2007; Ramus and Gayther, 2009). The HR-deficiency was also detected in BRCA1/2-proficient cells. Such a condition is currently defined as BRCAness of a tumor (Lord and Ashworth, 2016). The BRCAness phenotype is frequently found in breast, ovarian, pancreatic, prostatic, and other types of cancers (Alexandrov *et al.*, 2015; Holter *et al.*, 2015; Robinson *et al.*, 2015; Waddell *et al.*, 2015; Davies *et al.*, 2017) . Given the selective acquisition of BRCAness in tumors but not in healthy cells, therapeutic targets that specifically kill HR-deficient tumors but not proficient cells from patients were explored. Such a synthetic lethality (SL) approach has already transcended from academic laboratories to pharmaceutical companies. Poly [adenosine diphosphate (ADP)-ribosyl] polymerase inhibitors (PARPi) were developed to selectively increase cell killing of HR-deficient cancer cells sparing HR-proficient cells (Lord and Ashworth, 2017). Notably, four PARP inhibitors are already available for clinical use (Olaparib from AstraZeneca approved in 2015, Rucaparib from Clovis approved in 2016, Niraparib from TESARO/GSK approved in 2017, and Talazoparib from Pfizer approved in 2018). Moreover, the optimization of PARPi is still a subject of current research, and it is thus possible that other PARPi will be approved for clinical use in the near future (Sun *et al.*, 2018).

Despite their current success in the clinical setting, the mechanism of action of PARPi has only been partially elucidated. While it is accepted that double-strand breaks (DSBs) are frequently formed in BRCA-deficient cells, the trigger of such DNA lesions is a subject of debate. Originally, the SL induced by PARPi was attributed to the inhibition of base excision repair (BER) (Bryant *et al.*, 2005; Farmer *et al.*, 2005). PARPi-mediated inhibition of BER was proposed to trigger DSBs when unresolved single-strand breaks on DNA are encountered by replication forks in S-phase. Such replication-associated DSBs are expected to be toxic in the absence of HR. However, contrasting evidence showed that BER deficiency, and even PARP knockout, do not recapitulate the phenotypes caused by PARPi (Helleday, 2011). Instead, the trapping of the PARP enzymes in the regions of DNA damage seems to be the synthetic lethal event triggered by PARPi. When encountered by the replisome, such persistent PARP/DNA complex triggers stalling, collapse and breakage of replication forks into DSBs (Pommier *et al.*, 2016). Hence, both previous and current models suggest that the cause of SL triggered by PARPi depends on the accumulation of DSBs.

A different line of evidence demonstrates that replication-associated DSBs are formed after PARPi treatment. Chromosome aberrations generated at DSBs accumulate in BRCA-depleted samples treated with PARPi (Farmer *et al.*, 2005). Moreover, the genomic signature of BRCAness also requires DSB formation (Davies *et al.*, 2017). However, the evidence of DSB persistency so far accumulated in the literature is almost exclusively related to the analysis of cells with γH2AX, 53BP1, and Rad51 nuclear foci (Bryant *et al.*, 2005; Farmer *et al.*, 2005; Rottenberg *et al.*, 2008; Jaspers *et al.*, 2013; Johnson *et al.*, 2013; Michl *et al.*, 2016). Here we use an isogenic system to downregulate either BRCA1 or BRCA2 in colorectal cells, both of which showed strong sensitivity to Olaparib. As reported in other cellular models, the fraction of cells with numerous γH2AX and 53BP1 foci was upregulated in BRCA-depleted samples treated with Olaparib, and such replication stress was followed by chromosome instability. However, the neutral comet assay did not reveal a significant upregulation of DSBs in Olaparib-treated BRCA samples. Together, these results suggest that DSB formation but not its persistency precede both cell death and genomic instability in Olaparib treated BRCA-deficient cells.

## Material and Methods

### Cell lines and cell culture

HCT116^p21-/-^ cells were generated (Bunz *et al.*, 1998) and kindly provided by B. Vogelstein. Cell culture was performed in DMEM medium (Thermo Fisher Scientific) supplemented with 10% FBS (GIBCO-NZ) and 1% penicillin-streptomycin. Control for mycoplasma contamination was performed periodically with a PCR-based method with internal loading control. To generate cell lines expressing fluorescent proteins, all the fluorescent proteins were expressed from the same backbone from Clontech as described in Carbajosa *et al.* (2019). Briefly, transfection of vectors encoding fluorescent proteins (piRFP- C1, pECFP-C1, pmCherry-C1) was performed using JetPrime (Polyplus-transfection) according to manufacturer’s instructions. After multiple rounds of cell sorting (3-5) performed with FACS Aria II (BD bioscience), stable cell line pools expressing the different fluorescent proteins were established. The resulting cell lines pools were transduced with control, shBRCA1, and shBRCA2 using titers that promoted the higher downregulation BRCA1 and BRCA2 by qPCR and WB, yet keeping similar proliferation rates to the shSCR-transduced cell lines. Our goal here was to avoid clonal selection, which is often an issue that could result in misleading conclusions when generating stable cell lines. shSCR, shBRCA1, and shBRCA2 cell lines were used for experimentation for no more than six passages after the establishment of the cellular pools.

### DNA constructs and shRNA

shBRCA1 (TRCN0000010305, Sigma-Aldrich) and shBRCA2 (Carlos *et al.*, 2013)**,** were cloned into pLKO.1-TRC vector through *EcoR*I and *Age*I restriction sites; and shSCR-pLKO.1 was previously described (46). shSCR-plenti (TR30021), was acquired from Origene.

### Antibodies

Primary antibodies used were: α-BRCA1 (Oncogene Research), KU70 (Abcam), α γH2AX Ser 139, Upstate (Millipore, clone JBW301), α 53BP1 (Santa Cruz). Secondary antibodies used were Anti-mouse IgG (Sigma-Aldrich A 4416) for western blot analysis and α-mouse/rabbit-conjugated Cy2/Cy3 antibodies (Jackson Immuno Research) for immunofluorescence assays. Nuclei were stained with DAPI (Sigma).

### Cell counting methods

Cells were seeded on 96-well dishes at a density of 1500 cells/well. Cells were fixed with 2% paraformaldehyde/sucrose. InCell 2200® was used to obtain images of DAPI-stained nuclei and an InCell Analyzer WorkStation*®* was used to count nuclei. Alternatively, the number of viable HCT116 p21-/- shBRACA1/2 and shSCR cells was determined with a CellTiter-Glo® Luminescent Cell Viability Assay G-7570 (Promega), according to the manufacturer’s instructions. When assessing growth rates, cells stably expressing iRFP were seeded in 96-well plateat 2x10^3^cell/well and plates were scanned daily in the Odyssey Clx System (LI-COR Biosciences) as previously reported (Hock *et al.*, 2014). For flow cytometry experiments, isogenic cell populations were counted using a Countess FL device (Thermo Fisher) previous to plating the co-cultures.

### Co-cultivation method

Transfection protocols were performed using JetPrime (Polyplus-transfection) according to the manufacturer’s instructions. Multiple rounds of cell sorting (3-5) were performed (FACS Aria II, BD Bioscience) to select pools that express CFP, iRFP, and mCherry fluorescent proteins. Each “colored” cell line was sub-sequentially used to generate shSCR, shBRCA1, and shBRCA2 expressing pools (Carbajosa *et al.*, 2019). The transduced cells that showed the higher downregulation of BRCA1 and BRCA2 by qPCR and WB, yet keeping similar proliferation rates to the shSCR-transduced cell lines, were selected for survival assays. Equal amounts of cells were plated in a single 96 well ensuring ~33% distribution for each cell line. As a consequence of the equal proliferation rate, such distributions were conserved at the endpoint, which took place 6 days later. SL treatment alters the composition in a way that the proportion of shSCR increases and the shBRCA1 and shBRCA2 decrease.

### Cell cycle analysis

Cells were fixed with ice-cold ethanol and re-suspended in PBS containing RNase I (100 mg/mL, Sigma) and propidium iodide (50 mg/mL, Sigma). Samples were subjected to fluorescence-activated cell sorting (FACS, Calibur, Becton Dickinson), and data were analyzed using the Summit 4.3 software (DAKO Cytomation) as previously described (Federico *et al.*, 2016).

### Protein analysis

For direct western blot (WB) analysis, samples were lysed in commercial Laemmli buffer (Bio-Rad) contining the reducing agent 2-mercaptoethanol. ECL detection (Amersham, GE Healthcare) was performed according to the manufacturers’ instructions. Western blot images were taken with Image QuantLAS4000 (GE Healthcare), which allows capture and quantification of images within a linear range.

### Quantitative RT-PCR

Cells were lysed and total RNA was extracted using TRIzol® Reagent (Invitrogen). Reverse transcription was performed andBRCA2mRNA levels were measured with Fw-5’-AGGGCCACTTTCAAGAGACA3’ and Rv-5’TAGTTGGGGTGGACCACTTG3’ primers using the iQ SYBR Green Supermix (Invitrogen). Relative expression levels were normalized to GAPDH.

### Immunostaining and microscopy

Cells were fixed in 2% (w/v) paraformaldehyde (PFA)/2% sucrose and permeabilized with 0.1% (v/v) Triton X-100 in phosphate buffered saline (PBS). Blocking during 2 h at RT in PBS 2% (v/v) donkey serum (Sigma) was performed. Coverslips were incubated for 1 h in primary antibodies and then 1 h in secondary antibodies. Images were obtained with a Zeiss Axioplan confocal microscope or a Zeiss Axio Imager.A2. A total of 250-300 nuclei were analyzed per sample following the procedure described in (Mansilla *et al.*, 2016).

### Chromosomal aberration analysis

Metaphase chromosome spreads were generated introducing minor modifications to protocols previously used by us (Federico *et al.*, 2016). Before harvesting, cells were treated with Colcemid (0.08 μg/mL, KaryoMAX, Invitrogen) for 3 h. Cell pellets were incubated in hypotonic buffer (KCl 0.0075 M) at 37 °C for 4 min, followed by fixation in Carnoy’s fixative (3:1 methanol: glacial acetic acid). Cells were dropped onto slides and air-dried before staining with 6% (w/v) Giemsa in Sorensen’s buffer (2:1 67 mM KH2PO4 : 67 mM Na_2_HPO_4_, pH 6.8) for 2 min. 70 metaphase spreads were evaluated to detect gaps, breaks, and exchanges using an Applied Imaging Cytovision (2.10.17 version, Leica).

### Micronucleus (MN) assay

Cells seeded at low density were treated. Micronuclei analysis was performed using protocols previously described previously by us (Federico *et al.*, 2016). Samples were incubated with cytochalasin B (4.5 μg/mL, Sigma) for 36 h. Cells were washed for 1 min with hypotonic buffer (KCl 7.5 mM), twice with PBS and fixed with PFA/sucrose 2% for 20 min. DAPI staining served to visualize whole cells and nuclei respectively. 300 binucleated cells were analyzed and the frequency was calculated as MN/binucleated cells.

### Neutral comet assay

We used protocols previously described by Murfuni *et al.* (2013) with some modifications. Briefly, cells were embedded in 0.5% low-melting agarose on a slide and treated with a lysing solution (EDTA 30mM, SDS 0.5%) for 10 min at 4 °C. Slides were washed twice with deionized water (ddH_2_O), immersed in TBE 1X and subjected to electrophoresis at 17 V (6-7 mA) during 5 min at 4 °C. Samples were washed with ddH_2_O and stored in methanol overnight DNA was stained with propidium iodide and samples were examined with a Zeiss fluorescence microscope. To determine the tail moment (tail length x fraction of total DNA in the tail), 100-150 nuclei were evaluated per each condition using the OpenComet program.

### Statistical analysis

Statistical analyses were performed using GraphPad Prism 5.0 (GraphPad Software), applying the Student’s *t*-test and ANOVA test as appropriate. Graphs were generated using the same software.

## Results

### Downregulation of BRCA1 or BRCA2 levels sensitizes HCT116 colorectal cancer cells to Olaparib treatment

**Figure 1:**
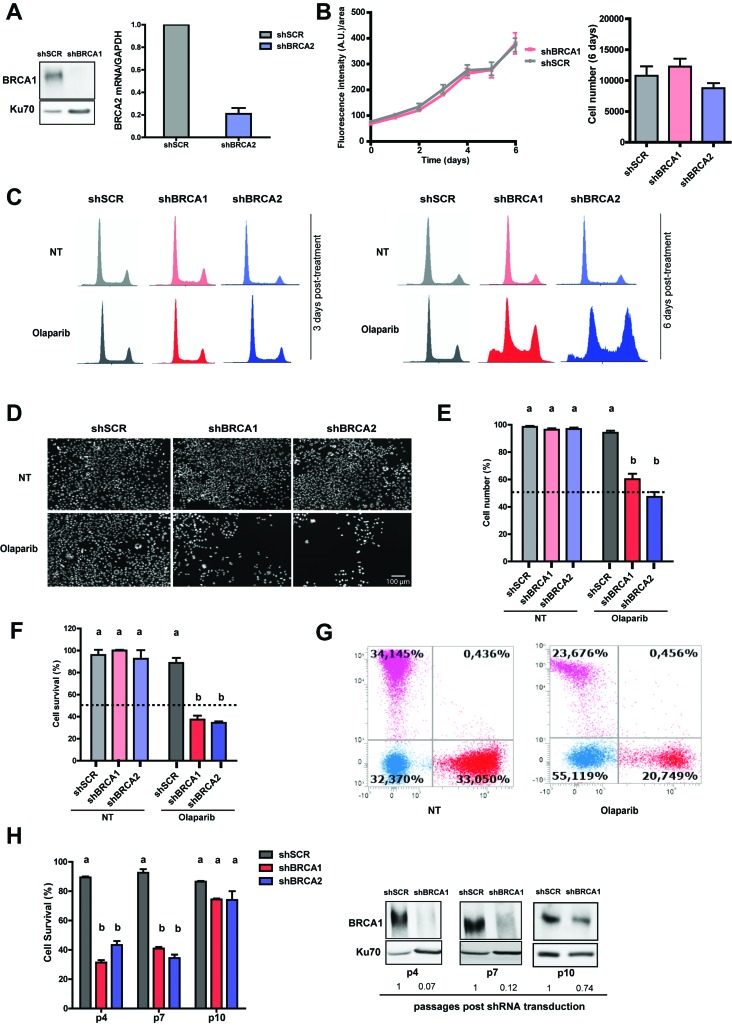
BRCA1 and BRCA2 downregulation sensitize colorectal cancer HCT116^p21-/-^ cells to Olaparib treatment. A) HCT116^p21-/-^ were transduced with control (shSCR_scramble) and shRNA vectors specific for BRCA1 and BRCA2 (shBRCA1 and shBRCA2). Western blot and RT-qPCR showing the levels of BRCA1 and BRCA2 protein and mRNA, respectively, at passage 4 after transduction. B) Growth curves of untreated samples. Data are shown as mean ± SD from 5 independent experiments. C) Flow cytometry of propidium iodide-stained samples. D) Representative panels depicting the SL induced in the HCT116^p21-/-^ cells after BRCA downregulation. Samples were stained with DAPI and photographs were obtained after automatic capture. E-F) HCT116 ^p21-/-^ cells transduced with shSCR and shRNA vectors specific for BRCA1 and BRCA2 (shBRCA1 and shBRCA2) were plated at a density of 1500 cells/well in 96 well plates. Six days later, samples were counted with direct (automatized cell counter-E) and indirect (F-cell titer Glo) methods. The plot shows the quantification (mean ± SD) of the surviving fraction of 3 independent experiments. G) HCT116^p21-/-^ cells were transduced with fluorescent proteins to generate colored pools as described in the material and methods section. After shRNA transduction, samples were co-cultured and treated with Olaparib when indicated. The plot shows the quantification of the surviving fraction of 3 independent experiments. H) Synthetic lethality (SL) is lost with increasing cell passages (mean ± SD, n= 2). Western blot showing BRCA1 levels at different times post shRNA transduction. Numbers below each lane are the quantification of normalized BRCA1 levels. Cell number was determined at the indicated passages and the SL was calculated. Statistical analysis was performed using two-way ANOVA with Bonferroni post-hoc test and differences were considered significant with *p* ≤ 0.001. The letters above the different values indicate groups that are significantly different.

Generating BRCA-deficient cell lines by stable knockdown is a difficult process, as most cells do not survive in the long term. The main limitation to reach stable BRCA depletion is the abrupt proliferation arrest that follows a severe knockdown or depletion of BRCA (Feng and Jasin, 2017). In order to achieve an efficient knockdown of BRCA proteins, we set up an shRNA-based downregulation of BRCA1 or BRCA2. Many cell lines did not recover after an acute crisis, and other cell lines regained BRCA expression after a few passages (Carbajosa *et al.*, 2019). We then explored colorectal cancer cells that are not characterized by a high frequency of BRCA loss. We chose a modified HCT116 cell line lacking the cyclin kinase inhibitor p21 that, therefore, has an attenuated G1/G2 checkpoint (Bunz *et al.*, 1998), which could favor the establishment of stable BRCA-deficient cells. It should be also mentioned that p21 deficiency negatively regulates BRCA2-mediated repair of replication-coupled DSBs (Mauro *et al.*, 2012), which could be relevant for controling BRCA1 deficient but not BRCA2 deficient cells. As described in the methods sections we set up a protocol that efficiently downregulated BRCA1 and BRCA2 (Figure 1A). In contrast to other cells lines tested, HCT116^p21-/-^ showed similar proliferation rates on BRCA-proficient and BRCA-deficient cells (Figure 1B). Flow cytometry analysis showed a strong BRCA1/2-related cell cycle arrest and subG1 accumulation in BRCA-depleted samples (Figure 1C). Synthetic lethality (SL) induction after Olaparib treatment was observed at 6 days post-Olaparib treatment in single cultivation methods and co-cultivation experiments (Figure 1D-G). The SL correlated with the efficiency of BRCA1 downregulation, reaching a critical level at passage 10 (Figure 1H). Hence, colorectal cancer cells, which do not frequently lose BRCA, can be sensitized to Olaparib by BRCA knockdown.

### Olaparib-triggered cell death in BRCA-deficient samples is preceded by the accumulation of markers of double-strand break formation and repair

**Figure 2:**
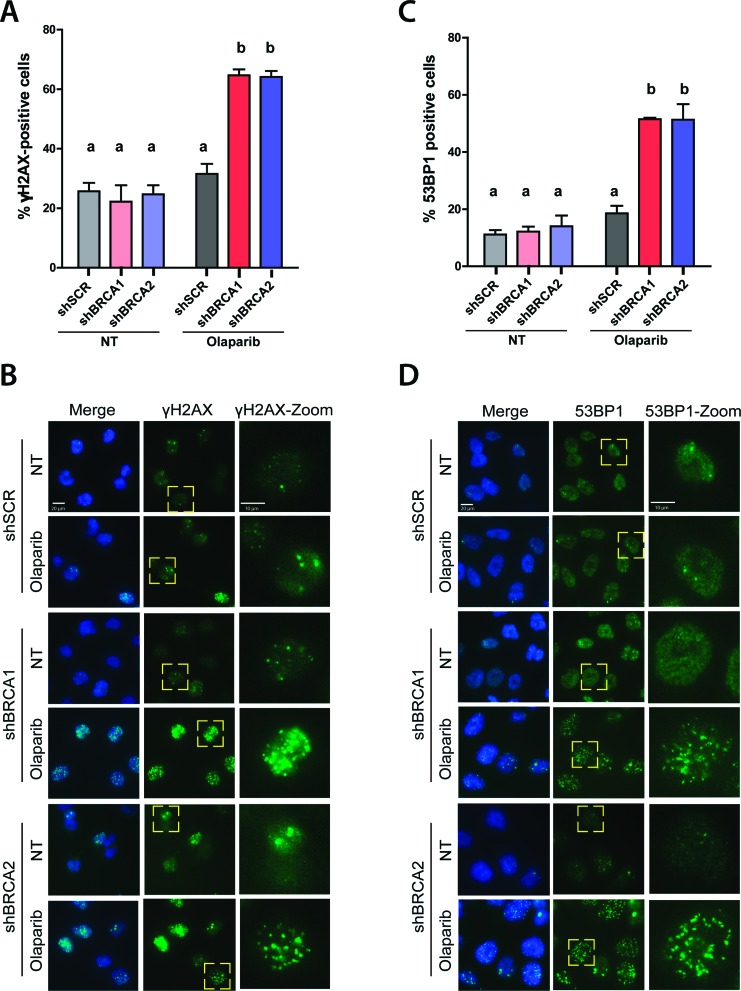
The number of cells with γH2AX and 53BP1 foci in BRCA-depleted HCT116^p21-/-^ cells increases after Olaparib treatment. A) HCT116^p21-/-^ cells transduced with shSCR, shBRCA1, and shBRCA2 cells were treated with Olaparib. After 48 h, immunostaining with γ-H2AX antibodies was performed. The percentage of cells with foci was quantified using fluorescence microscopy (magnification: 100X). Nuclei with more than 35 γH2AX focal structures were considered positive. At least 300 cells per condition were analyzed in 5 independent experiments. Statistical analysis was performed using Two-way ANOVA with Bonferroni post-hoc test (***p ≤ 0.001). Data are shown as mean ± SD. B) Representative images of data showed in A. Zoom images of the nuclei indicated with the yellow dotted square are showed on the left. C) HCT116^p21-/-^ shSCR and shBRCA1 cells were treated with Olaparib. After 48 h, immunostaining with a 53BP1 antibody was performed. The percentage of cells with foci was quantified using fluorescence microscopy (magnification: 100X). Only nuclei with more than five 53BP1 foci were quantified as positive. At least 300 cells per condition were analyzed and data are shown as mean ± SD from5 independent experiments. D) Representative images of data showed in C. Zoom images of the nuclei indicated with the yellow dotted square are showed on the left. Statistical analysis was performed using Two-way ANOVA with Bonferroni post-hoc test and differences with *p* ≤ 0.001 were considered significant. In all graphs, the letters above the different values indicate groups that are significantly different.

Many reports indicate that the treatment of BRCA-deficient cells with PARPi triggers an acute increase of replication stress that leads to the accumulation of DSBs. Such DSBs were frequently revealed as γH2AX foci formation in the nucleus of PARPi-treated cells (Bryant *et al.*, 2005; Farmer *et al.*, 2005; Rottenberg *et al.*, 2008; Jaspers *et al.*, 2013; Johnson *et al.*, 2013; Michl *et al.*, 2016). In our experimental settings, the percentage of cells with high levels of γH2AX foci significantly increased at 2 days after Olaparib treatment (Figure 2A,B), which is in agreement with previous reports. The percentage of cells with γH2AX decreases after that time, reaching levels that are similar to those of untreated conditions at 6 days (not shown). The localization of 53BP1 to nuclear foci also increased in the same experimental conditions and at 48 hours after Olaparib treatment (Figure 2C,D). Such observations suggested that in HCT116^p21-/-^ cells depleted from BRCA proteins, acute replication stress precedes the cell death triggered by PARPi. Moreover, the rapid recruitment of 53BP1 to such DSBs indicates that DSBs could be rapidly repaired by a 53BP1-driven repair.

### Olaparib-triggered cell death in BRCA-deficient HCT116^p21-/-^ is preceded by accumulation of chromosome instability

**Figure 3:**
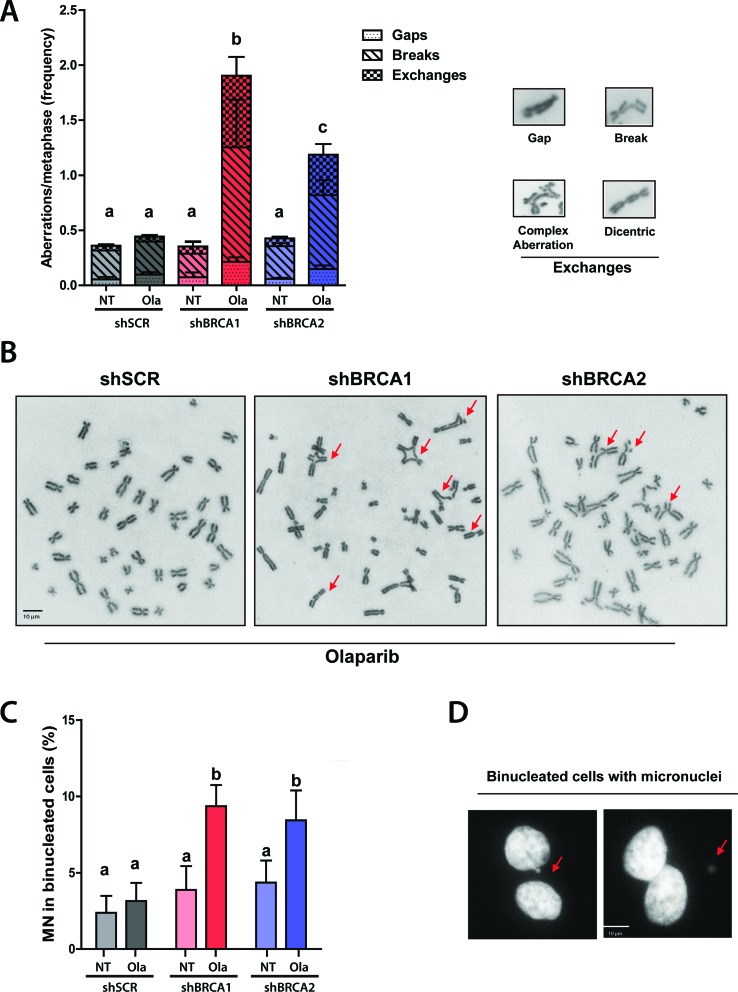
Chromosome instability precedes Olaparib-triggered cell death in BRCA-deficient HCT116^p21-/-^. A) HCT116^p21-/-^ cells transduced with shSCR, shBRCA1 and shBRCA2 were submitted to cytogenetic analysis 48 h after the treatment with Olaparib. The frequency of gaps, breaks, and exchanges was calculated after analyzing a minimum of 70 metaphases per condition in 5 independent experiments. B) Representative images of chromosomic aberrations quantified in A. C) HCT116^p21-/-^ transduced with shSCR, shBRCA1 and shBRCA2 were treated for 24 h with Olaparib and were arrested at a binucleated stage using cytochalasin B. The frequency of micronuclei was estimated (shown as mean ± SD, using DAPI staining and fluorescence microscopy (magnification: 100X), analyzing a minimum of 300 binucleated cells per condition in 4 independent experiments. D) Representative images of chromosomic aberrations quantified in C. Statistical analysis was performed using two-way ANOVA with Bonferroni post-hoc test and differences were considered significant with *p* ≤ 0.001. The letters above the different values indicate groups that are significantly different.

In the context of BRCA-depletion, 53BP1 favors the repair of DSBs by non-homologous end joining (NHEJ) (Daley and Sung, 2014). Since PARPi-induced DSBs are actually one-ended DSBs formed at the tip of collapsed replication forks, the NHEJ-mediated processing of such DSBs indefectible causes formation of radial chromosomes and increase other types of chromosome instability (Federico *et al.*, 2018). In agreement with results obtained in mammary and ovarian models, the depletion of BRCA proteins in HCT116^p21-/-^ colorectal cancer cells cause massive genomic instability after Olaparib treatment. Such genomic instability was manifested as the extensive accumulation of gaps, breaks, radial chromosomes (Figure 3A,B) and micronuclei (Figure 3C,D), which are all markers of aberrant repair of DSBs (Federico *et al.*, 2018). Together these experiments show that a steep increase in genomic instability temporally precedes cell death in PARPi-treated HCT116^p21-/-^ cells.

### Olaparib-triggered cell death in BRCA-deficient samples is not preceded by persistent double-strand breaks

**Figure 4:**
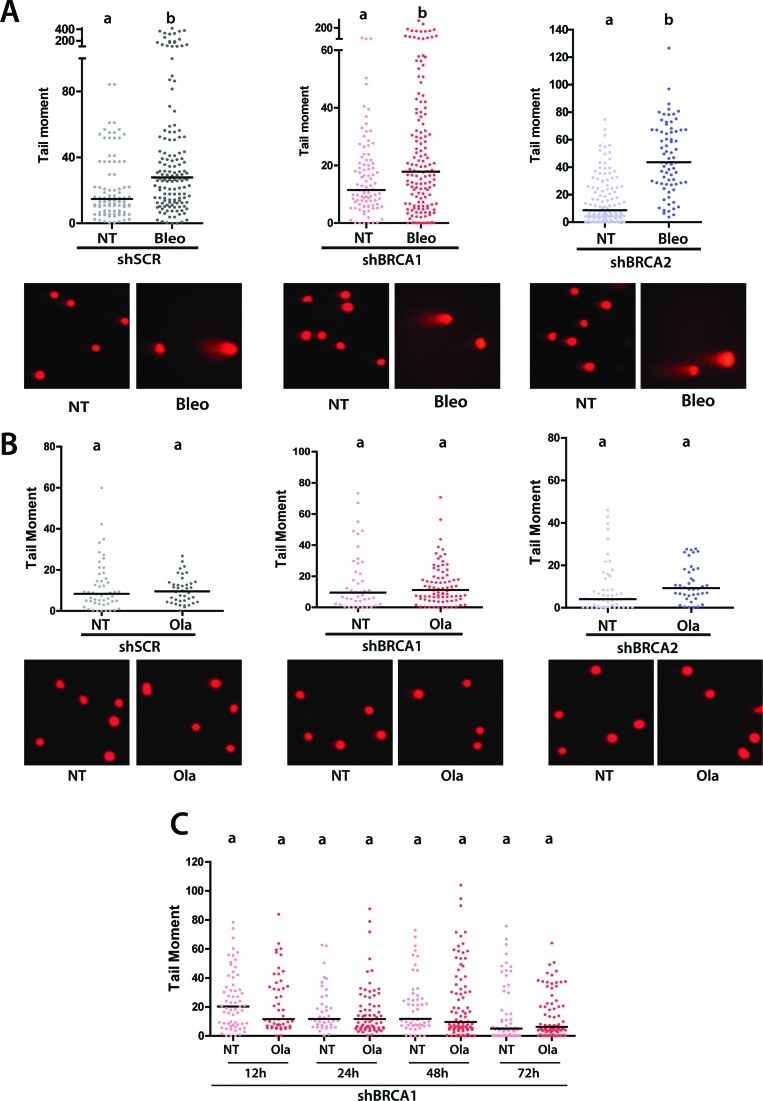
Persistent double-strand break accumulation does not precede Olaparib-triggered cell death in BRCA-deficient HCT116^p21-/-^ cells. A) HCT116^p21-/-^ cells transduced with shSCR, shBRCA1 and shBRCA2 were submitted to neutral comet assay in untreated and Bleomycin-treated conditions (mean +SD, n= 2). B) HCT116^p21-/-^ cells transduced with shSCR, shBRCA1 and shBRCA2 were submitted to neutral comet assay two days after Olaparib treatment, which coincides with the maximum levels of γH2AX accumulation. Results are representative of 2 independent experiments. C) HCT116^p21-/-^ cells transduced with shSCR and shBRCA1 were submitted to neutral comet assay at the indicated days after Olaparib treatment. Results are representative of 2 independent experiments. Statistical analysis was performed using One Way ANOVA with Dunns comparison test and differences were considered significant with *p* ≤ 0.05. The bars on top of the distribution clouds indicate the median. The letters above the different values indicate groups that are significantly different.

**Figure 5:**
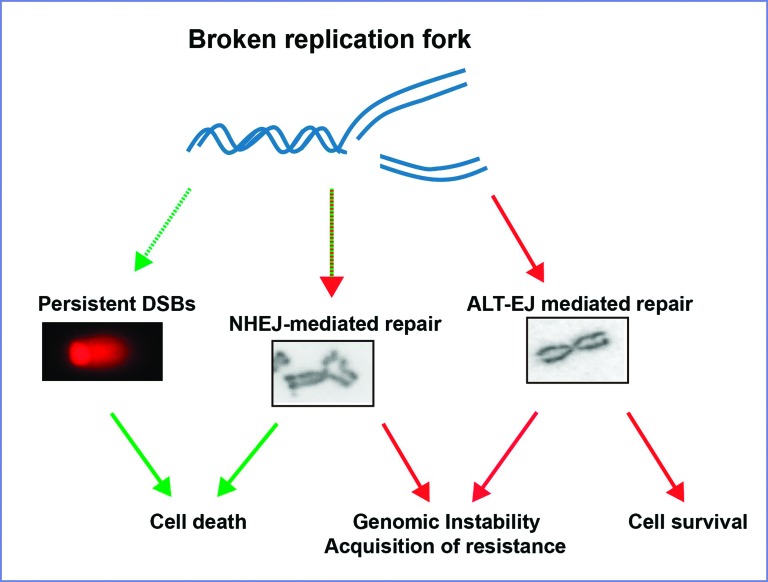
Model depicting the implications of low DSB accumulation after PARPi in BRCA-deficient cells. In BRCA-deficient cells, Olaparib treatment causes replication fork collapse. In rare samples, DSBs may accumulate and such a persistent load of DSBs may trigger cell death (left outcome). However, in most samples, DSBs are processed by error-prone pathways, such as non-homologous end joining (NHEJ) and alternative end joining (ALT-EJ). Error-prone pathways such as ALT-EJ, may not induce aberrant re-arrangement of chromosomes and may promote cell survival (middle outcome - see discussion). Other error-prone pathways that cause chromosome re-arrangement correlate with decreased cell survival (right outcome - see discussion).

While the accumulation of cells with γH2AX foci is accepted as a marker of DSB accumulation in many PARPi-related studies, experts in the field have addressed the limitations of such markers (Zellweger *et al.*, 2015). Intriguingly, the maximum percentage of cells with γH2AX foci was observed at 2 days after Olaparib treatment (Figure 2B) although cell death was negligible even at 3 days after Olaparib treatment (Figure 1C). Hence, we wondered whether DSBs formed at the time of maximal γH2AX detection would accumulate for a long time to eventually trigger cell death days later. We reasoned that direct detection of DSBs should be set up, and so we optimized the neutral comet assay to be used in our experimental conditions. Bleomycin was used as a positive control to observe the accumulation of DSBs in HCT116^p21-/-^ cells (Figure 4A). Surprisingly, DSBs were not detected by neutral comet assay after Olaparib treatment in both BRCA-deficient cell lines (Figure 4B,C). Ruling out the possibility of a delayed accumulation of DSBs, the neutral comet assay did not reveal DSBs at any time points after Olaparib treatment (Figure 4C). Together, these results demonstrate that persistent accumulation of DSBs is not frequent in Olaparib-treated BRCA-deficient HCT116^p21-/-^cells. Hence, cell death is unlikely triggered by unrepaired DSBs in these settings. Instead, DSBs may indirectly trigger cell death in a manner that depends on the accumulation of unstable chromosomes generated by dysregulated error-prone pathways (Figure 5). Together, these results show that genomic instability and cell death are intimately associated with BRCA-deficient HCT116^p21-/-^ cells treated with PARPi. The implications of these findings for the acquisition of resistance in PARPi treated BRCA cancers will be discussed below.

## Discussion

The accumulation of unrepaired DSBs is considered to be the trigger of cell death in BRCA-deficient cells treated with PARPi (Bryant *et al.*, 2005; Farmer *et al.*, 2005). However, at least in the isogenic cellular model presented in this study, BRCA-depletion triggered cell death and chromosome instability but not detectable DSB accumulation. Such a finding suggests that the cause of cell death in this scenario is not mainly associated with the persistence of unrepaired DSBs, but with the accumulation of other triggers of cell death, possibly resulting from the type of DNA repair pathway chosen for DSB repair.

### DSBs repair pathway choice as a trigger of cell death in PARPi-treated BRCA1/2 deficient cells

The acquisition of resistance to PARPi has been mainly linked to the dysregulated activation of error-prone repair pathways in BRCA-deficient cells (Lord and Ashworth, 2013). However, other mechanisms of resistance were also described. An indirect mechanism reported was the increased expression of genes that encode for the drug efflux transporter P-glycoprotein (Rottenberg *et al.*, 2008). Furthermore, the recovery of HR capacity (e.g., reversion of primary mutations or secondary mutations that restore BRCA function) promotes resistance to Olaparib (Edwards *et al.*, 2008; Sakai *et al.*, 2008; Swisher *et al.*, 2008; Norquist *et al.*, 2011). The loss of proteins that facilitate NHEJ activation, such as 53BP1, also promote resistance to PARPi and restoration of HR (Bunting *et al.*, 2010; Jaspers *et al.*, 2013). PTEN and Rev7 loss were additionally described as mechanisms of resistance driven by HR restoration (Peng *et al.*, 2014; Xu *et al.*, 2015). Besides the recovery of HR, stabilization of the replication fork by a limitation of exonuclease activity of MRE11 was reported as a trigger for PARPi resistance in BRCA-deficient backgrounds (Ray Chaudhuri *et al.*, 2016; Rondinelli *et al.*, 2017). Hence, PARPi resistance is linked to both the amount of DSBs formed and the loss of HR.

There is much less available evidence supporting that the dysregulation in DSBs repair also influences the extent of cell death caused by PARPi. The choice of alternative end joining (Alt-EJ) prevents cell death in PARPi-treated BRCA1-deficient cells (Ceccaldi *et al.*, 2015). Conversely, the elimination of pol θ prevents ALT-EJ and increases cell death (Ceccaldi *et al.*, 2015). It is currently unknown whether such cell death is associated with the accumulation of persistent DSBs, or whether it depends on the HR-independent resolution of DSBs. Another factor to take into consideration when evaluating variables that influence the survival of BRCA-deficient cells treated with PARPi is fork stabilization. ALT-EJ promotes fork stabilization and prevents its excessive processing (Kais *et al.*, 2016). Hence, the repair of DSBs via ALT-EJ promotes the survival of BRCA-deficient cells treated with PARPi. Intriguingly, the NHEJ-mediated repair of DSBs has the opposite effect on cell death induction. As a consequence of HR restoration, 53BP1 and Rev7 loss increases cell survival of PARP treated cells (Bunting *et al.*, 2010; Jaspers *et al.*, 2013; Xu *et al.*, 2015). It is therefore difficult to establish a causal link between chromosome instability and cell death. However, there are several correlations that put such a case into consideration. For example, FANCD2 loss increases both cell death and chromosome instability of BRCA2-deficient cells (Michl *et al.*, 2016). In addition, EZH2 depletion induces survival and prevents genomic instability in BRCA2-deficient tumors in a manner that depends on Mus81 loading to replication forks (Rondinelli *et al.*, 2017). PTIP loss also reduces both cell death and chromosome instability in BRCA1-deficient samples treated with PARPi, in this case without restoring HR (Ray Chaudhuri *et al.*, 2016). Moreover, expression of the micro RNA miR-493-5p affects the survival of PARPi-treated BRCA2-mutated/depleted cells by modulating the levels of nucleases involved in maintaining genomic stability and without affecting HR (Meghani *et al.*, 2018). Therefore, chromosome instability and cell death concomitantly occur in PARPi treated BRCA-deficient cells. As chromosome instability temporally precedes cell death it could be proposed that the trigger for cell death is a toxic upregulation of chromosomic instability. In fact, in the context of ATM deficiency, PARPi also induces cell death, which has been attributed to the accumulation of toxic NHEJ-generated aberrant chromosomes (Balmus *et al.*, 2019). Intriguingly, in such experimental settings, ATM-deficient cells do not accumulate DSBs, as revealed by a neutral comet assay (Balmus *et al.*, 2019). Hence, as in ATM-deficient cells, BRCA-deficient cells may die because of toxic chromosome instability triggered by PARPi.

### Proofs of DSBs accumulation in BRCA-deficient cells treated with PARPi

Few assays can directly reveal DSBs, and such assays may not be very sensitive to low levels of DSBs. However, both pulse field gel electrophoresis (PFGE) and neutral comet assays can reveal DSBs reported to take place in HR-proficient settings (Elvers *et al.*, 2012; Murfuni *et al.*, 2013; Federico *et al.*, 2016; Quinet *et al.*, 2016). Such results suggest that the assays should be sensitive enough to detect DSBs in the context of HR-deficient cells. Intriguingly, such techniques were only rarely applied to BRCA-deficient cells treated with PARPi (Clements *et al.*, 2018; Gogola *et al.*, 2018). To our knowledge, this is the first report that uses a direct DSB detection method to explore the effect of the PARPi treatment on BRCA-depleted cells (without the addition of other genotoxins). Instead of using direct DSBs detection, the PARPi field has chosen to focus its attention on the accumulation of γH2AX nuclear foci as a marker of DSBs. Whether γH2Ax nuclear foci strictly correlate with DSBs accumulation is a subject of debate. Some laboratories have attempted to combine it with 53BP1 colocalization and to obtain independent evidence of DSBs formation, such as the accumulation of pATM and pKap1 (Berti *et al.*, 2013; Zellweger *et al.*, 2015; Federico *et al.*, 2016; Perkhofer *et al.*, 2017). However, the proof of DSBs formation is strictly dependent on the detection of such DNA lesions in PFGE or neutral comet assays. Multiple lines of evidence demonstrate that DSBs are formed after the PARPi treatment of BRCA-deficient samples. Most chromosome aberrations, as well as micronuclei, can only be formed from DSBs (Federico *et al.*, 2018). Moreover, the BRCAness signature is also associated with DSBs formation (Davies *et al.*, 2017). That being said, our data indicate that while DSBs are generated, they are not persistent enough to be the trigger for cell death as suggested by the currently accepted mode of action of PARPi. We hypothesize that DSBs are frequently formed but rapidly repaired by end-joining-mediated pathways after PARPi treatment. Perhaps in the future this hypothesis could be further evaluated by the modification of methods such as the NHEJ host reactivation assay, which needs to be adjusted according to the characteristics of PARPi-triggered DSBs (Nagel *et al.*, 2014). At least in this scenario, γH2AX nuclear foci may rather reveal sites where DSB repair has occurred and sites of unresolved DSBs. These findings, therefore, suggest caution in the interpretation of γH2AX foci data, a limitation that may extend to the analysis of other nuclear foci in the DNA damage response field.
